# Clinical association of metabolic syndrome, C‐reactive protein and testosterone levels with clinically significant prostate cancer

**DOI:** 10.1111/jcmm.13994

**Published:** 2018-11-18

**Authors:** Enrique Gómez‐Gómez, Julia Carrasco‐Valiente, Juan Pablo Campos‐Hernández, Ana Maria Blanca‐Pedregosa, Juan Manuel Jiménez‐Vacas, Jesus Ruiz‐García, Jose Valero‐Rosa, Raul Miguel Luque, María José Requena‐Tapia

**Affiliations:** ^1^ Maimonides Institute of Biomedical Research of Cordoba (IMIBIC) Cordoba Spain; ^2^ Department of Urology Reina Sofia University Hospital Cordoba Spain; ^3^ Department of Cell Biology, Physiology and Immunology University of Cordoba (UCO) Cordoba Spain; ^4^ CIBER Physiopathology of Obesity and Nutrition (CIBERobn) Madrid Spain

**Keywords:** C‐reactive protein, inflammation, metabolic syndrome, significant prostate cancer, testosterone

## Abstract

Recently, the influence that metabolic syndrome (MetS), hormonal alterations and inflammation might have on prostate cancer (PCa) risk has been a subject of controversial debate. Herein, we aimed to investigate the association between MetS‐components, C‐reactive protein (CRP) and testosterone levels, and the risk of clinically significant PCa (Sig‐PCa) at the time of prostate biopsy. For that, men scheduled for transrectal ultrasound guided biopsy of the prostate were studied. Clinical, laboratory parameters and criteria for MetS characterization just before the biopsy were collected. A total of 524 patients were analysed, being 195 (37.2%) subsequently diagnosed with PCa and 240 (45.8%) meet the diagnostic criteria for MetS. Among patients with PCa, MetS‐diagnosis was present in 94 (48.2%). Remarkably, a higher risk of Sig‐PCa was associated to MetS, greater number of MetS‐components and higher CRP levels (odds‐ratio: 1.83, 1.30 and 2.00, respectively; *P* < 0.05). Moreover, higher circulating CRP levels were also associated with a more aggressive Gleason score in PCa patients. Altogether, our data reveal a clear association between the presence of MetS, a greater number of MetS‐components or CRP levels >2.5 mg/L with an increased Sig‐PCa diagnosis and/or with aggressive features, suggesting that MetS and/or CRP levels might influence PCa pathophysiology.

## 1 INTRODUCTION

Prostate cancer (PCa) is the most common cancer among men in developed countries and a leading cause of mortality and morbidity globally.^1^ The non‐modifiable risk factors established for PCa are age, race and family history,^2^ however, the contribution that lifestyle and environmental factors may have on PCa aetiology has been recently suggested, and certainly is still an active subject of debate.^3^, ^4^ In this sense, metabolic syndrome (MetS) is a widely prevalent disorder whose diagnosis consists on a combination of clinical and serological parameters including obesity (particularly abdominal adiposity), insulin resistance, elevated blood pressure, elevated triglyceride levels and decreased levels of high density lipoproteins (HDL)‐cholesterol.^5^

Several mechanisms have been proposed to explain the association between PCa and MetS including hormonal alterations (eg low circulating levels of testosterone), insulin resistance (eg high insulin and IGF‐1 levels) and inflammation status (eg alterations in cytokines and C‐reactive protein [CRP] levels, among others inflammatory‐related molecules).^6^ In this sense, we have recently uncovered the existence of a fine, germane crosstalk between the endocrine‐metabolic status and the development and homoeostasis of the prostate gland, wherein key components of the insulin, IGF1 and adipokines axes, among other, could play a relevant pathophysiological role.^7^, ^8^ In addition, it has been suggested that low levels of testosterone could be linked with the presence of abdominal obesity, and this in turn, might cause an alteration in the metabolism of fatty acids promoting insulin resistance,^9^ which might be associated to PCa risk^10^, ^11^; however, the association between circulating testosterone levels, metabolic status and PCa progression/aggressiveness remains controversial.^12^, ^13^, ^14^, ^15^ Furthermore, circulating levels of CRP, one of the most useful markers to assess varying degrees of inflammation in disease states such as obesity, diabetes mellitus (DM), etc.,^16^ have been found to be elevated in patients with different cancer types compared to healthy patients^17^; but the putative association between CRP levels, metabolic status, testosterone and PCa remains still unknown.^17^

Therefore, based on the information mentioned above, the aim of this study was to explore the associations and clinical consequences that the inflammatory status (using CRP levels), testosterone levels and MetS may have on the diagnosis and aggressiveness of PCa using a cohort of patients with and without MetS and/or PCa.

## 2 PATIENTS AND METHODS

### 2.1 Population

This is an observational study over an 18‐month prospective cohort, in patients who underwent ultrasound guided prostate biopsy. The study was carried out within a project approved by our Hospital Research Ethics Committee, and informed consent was obtained from all participants. Specifically, blood sample was obtained in the morning (between 8:00 and 10:00 am) after an overnight fasting and then, the prostate biopsy was implemented according to clinical practice. The inclusion criteria for this study was the indication of the biopsy by the clinician according to clinical practice. Recommendations to obtain a biopsy were the following: (a) in the case of non‐previous biopsy, suspicious findings on digital rectal examination (DRE), PSA >10 ng/mL, or PSA 3‐10 ng/mL if free PSA ratio was low (usually, <25‐30%), and (b) in the case of patients with previous biopsies with persistently suspicious of PCa (ie elevated PSA, suspicious DRE, etc.). On the other hand, the exclusion criteria were: (a) wait circumference or other relevant clinical data not well‐reported; (b) previously known PCa diagnosis, and (c) patients with acute infectious disease (not underwent prostate biopsy at this time).

### 2.2 Clinical data

Demographics information and medical histories of each patient were obtained. Specifically, information of previous diagnoses of hypertension, DM and hypercholesterolaemia was collected, as well as family history of PCa and current usage of 5α‐reductase inhibitors, metformin or statins. Moreover, each patient underwent a physical examination before the biopsy was carried out, including data of body weight (kg), height (cm) and waist circumference (cm). Specifically, the waist circumference was obtained by measuring the abdominal girth midway between the lowest rib margin and iliac crest while the patients were in a standing position.

As mentioned above, a blood sample (10 mL) was also collected after an overnight fasting period of ~8 hours. Levels of CRP (mg/L, by an Immunoturbimetric, High Sensitivity method; Ref. 6k26‐30/41; Abbott), testosterone (ng/mL, by a Chemiluminescent Microparticle Immunoassay method [CMIA]; Ref. 7k73; Abbott), PSA (ng/mL, by a CMIA; Ref. 7k70; Abbott), HDL (mg/dL by an accelerator selective detergent method; Ref. 3k33‐20; Abbott), triglycerides (mg/dL by a Glycerol Phosphate Oxidase method; Ref. 7D74‐20; Abbott), glucose (mg/dL, by a Hexokinase/G‐6‐PDH method; Ref. 3L82‐20 and 3L82‐40) and glycated haemoglobin (HbA1c; %, by HPLC; Bio‐Rad, Ref. 270‐2000) were measured following the manufacturer's instructions.

MetS status of each patient was evaluated according to the National Cholesterol Education Program Expert Panel on Detection, Evaluation and Treatment of High Blood Cholesterol in Adults, Adult Treatment Panel III criteria (ATP III).^18^ For the diagnosis of MetS, at least three of the following criteria had to be met:

1. Waist circumference >102 cm (>40 inches).
2. HDL cholesterol levels <40 mg/dL (<1.0 mmol/L) or being actively treated for low HDL levels.
3. Serum triglycerides levels ≥150 mg/dL (≥1.7 mmol/L) or being actively treated for elevated triglycerides.
4. Fasting glucose levels ≥100 mg/dL (≥6.1 mmol/L) or being actively treated for hyperglycaemia.
5. Diagnosis of elevated blood pressure or being actively treated for hypertension.

### 2.3 Prostate biopsy and pathologic analysis

Transrectal prostate biopsy was carried out under local anaesthesia using a standard peri‐prostatic block, a transrectal ultrasound transducer, and an 18G automated needle biopsy instrument. Usual recommendations were to take 12 cores in patients undergoing the first biopsy procedure, and a minimum of 16 biopsy cores for those who had a previous biopsy. As recently reported,^19^ all biopsy specimens were analysed by an expert urologic anatomo‐pathologist according to ISUP 2005 modified criteria.^20^

### 2.4 Statistical analysis

A descriptive study was performed by calculating the median and interquartile ranges for the quantitative variables and the absolute frequencies and percentages for the qualitative variables. The primary end‐point of the study was the presence of a clinically Sig‐PCa on biopsy. The tumours with a Gleason Score (GS) ≥7 were considered clinically Sig‐PCa. The MetS variables were assessed in a dichotomous manner according to whether 3 or more of the ATP III diagnostic criteria were met, and quantitatively based on the absolute number of criteria met. The age, PSA levels and biopsy number were categorized (ie age [<60, 60‐70 and >70 years], PSA [<3, 3‐10, 10‐20 and >20 ng/mL], and biopsies [1° or ≥2°]) to perform a multivariate analysis.

A Student's *t*‐test was used for analysis of the quantitative data and a chi‐squared test was used for the qualitative variables. A Pearson test was used to study the correlation between the quantitative variables. A receiver operating characteristic (ROC) curve analysis was performed to determine the best CRP levels cut‐off for the diagnosis of Sig‐PCa. Uni‐ and multivariate analyses were performed by logistic regression models to evaluate the association of the variables with PCa and Sig‐PCa. ROC curve analysis was also performed to determine the predictive capability of the variables together in the total cohort and, a sub‐analysis was also performed in patients with PSA <10 ng/mL. The De‐long test was used to compare the area under the curve (AUC) values.

A <5% level of significance was used to decide statistically significant differences to make our conclusions comparable to those of the related research. All the analyses and graphics were performed with GraphPad prism 6, MedCalc statistical software and SPSS version 17.0.

## 3 RESULTS

### 3.1 Population description

Clinical data of 655 patients were selected to be included in this study; however, 131 patients were excluded based on the criteria mentioned above. Therefore, a total of 524 patients were finally included in the analysis. The demographic and clinical data from this cohort of patients according to the MetS status are shown in Table 1. Specifically, 240 of the patients (45.8%) satisfied the diagnostic criteria of MetS (n = 94 were diagnosed with PCa [39.2%] and, from those, n = 54 [57.4%] had Sig‐PCa [GS ≥7]), while 284 of the patients had no MetS (n = 101 with PCa [35.6%] and, from those, n = 43 [42.5%] with GS ≥7) (Table 1). No statistical difference in family history of PCa, positive DRE or PSA levels were found between patients with or without MetS. However, patients with MetS were older, tended to have slightly higher prostate volume, had higher BMI, waist circumference, as well as elevated triglycerides, glucose and CRP levels but lower levels of HDL and testosterone (Table 1). A strong correlation between BMI and waist circumference was observed (*r* = 0.87; *P* < 0.001). Notably, the rate of Sig‐PCa diagnoses was significantly higher in patients with MetS compared with patients without MetS (*P* = 0.03). Moreover, within the patients with MetS, waist circumference, glucose levels and hypertension were the most common criteria for the diagnosis of MetS (ie 205, 191 and 188 patients of 240, respectively; >75% of the patients with MetS; Table 1).

**Table 1:** Descriptive and comparative analysis of demographics and clinical variables according to the presence or not of MetS

Variable	MetS (n = 240)	No MetS (n = 284)	*P*‐value	Total (n = 524)
Age; years old	66 (60‐70)	64 (58‐69)	0.01	65 (59‐70)
Family History; yes	35 (14.6)	52 (18.3)	0.29	87 (16.6)
Positive DRE; yes	51 (21.3)	57 (20.1)	0.74	108 (20.6)
Serum PSA; ng/mL	5.6 (3.8‐8.3)	5.8 (4.0‐8.4)	0.43	5.7 (3.8‐8.4)
5 alpha inhibitors	11 (4.6)	10 (3.5)	0.66	21 (4)
aProstate volume; cm^3^	39 (27‐54)	34.2 (26‐48)	0.06	35 (26‐51)
BMI; kg/m^2^	30.5 (28.2‐33.3)	26.8 (25.0‐29.0)	<0.01	28.4 (26.2‐31.3)
Waist circumference; cm	109 (104‐116)	99 (93.5‐104.5)	<0.01	103 (97‐111)
HDL; mg/dl	41 (35‐46)	47 (42‐55)	<0.01	44 (39‐51)
Triglycerides; mg/dl	135 (95‐176.8)	91 (74‐115)	<0.01	106 (79‐147)
Glucose; mg/dl	111 (100‐129)	94 (87‐101)	<0.01	100 (90‐113.5)
Metformin; yes	57 (23.8)	9 (3.2)	<0.01	66 (12.6)
Statin: yes	124 (51.7)	44 (15.5)	<0.01	168 (32.1)
HbA1c; %	5.8 (5.5‐6.2)	5.4 (5.1‐5.6)	<0.01	5.5 (5.2‐5.9)
CRP; mg/L	2.6 (1.4‐4.8)	1.7 (0.9‐4.1)	0.05	2.0 (1.1‐4.4)
Testosterone; ng/mL	4.4 (3.5‐5.7)	5.4 (4.4‐6.7)	<0.01	5.04 (3.97‐6.2)
MetS criteria				
Criteria I MetS	205 (85.4%)	86 (30.3%)	<0.01	291 (55.5)
Criteria II MetS	183 (76.3%)	81 (28.5%)	<0.01	264 (50.4)
Criteria III MetS	103 (42.9%)	26 (9.2%)	<0.01	129 (24.6)
Criteria IV MetS	191 (79.6%)	81 (28.5%)	<0.01	272 (51.9)
Criteria V MetS	188 (78.3%)	94 (33.1%)	<0.01	282 (53.8)
PCa; yes	94 (39.2%)	101 (35.6%)	0.42	195 (37.2)
Gleason Score ≥7; yes	54 (22.5%)	43 (15%)	0.03	97 (18.5)

BMI, body mass index; CRP, C‐reactive protein; DRE, digital rectal examination; HbA1c, glycated haemoglobin; HDL, high density lipoprotein; PCa, prostate cancer; MetS, metabolic syndrome [Criteria: I. Waist circumference > 102 cm (> 40 in); II. HDL cholesterol levels <40 mg/dL (<1.0 mmol/L), or being actively treated for low HDL levels; III. Serum triglycerides levels ≥150 mg/dL (≥ 1.7 mmol/L), or being actively treated for elevated triglycerides; IV. Fasting glucose levels ≥100 mg/dL (≥ 5.55 mmol/L), or being actively treated for hyperglycaemia, and; V. Diagnosis of elevated blood pressure or being actively treated for hypertension].

Values are expressed in median and interquartile range for quantitative variables and in absolute number and percentage for qualitative variables. Statistical test: *t*‐Student for quantitative variables and chi‐squared for qualitative ones.

a n = 441 patients (No MetS = 236 and MetS = 205).

### 3.2 Relationship between metabolic syndrome and circulating testosterone and CRP levels

Circulating levels of testosterone and CRP were analysed in the whole cohort of patients according to the individual diagnostic criteria of MetS (I, II, III, IV and V; Table 2). Interestingly, testosterone levels were significantly lower in patients that individually met each criterion of MetS compared to those that did not meet these criteria. In contrast, only patients that met the criterion I had higher CRP levels (Table 2).

**Table 2:** Association between circulating C‐ reactive Protein and testosterone levels with each of the criterion (I, II, III, IV or V) of MetS

MetS criteria	C‐reactive protein (mg/L)	*P*	Testosterone (ng/mL)	*P*
Criterion I				
Yes	2.7 (1.4‐5.2)	<0.01	4.5 (3.6‐5.8)	<0.01
No	1.5 (0.8‐3.4)		5.5 (4.4‐6.8)	
Criterion II				
Yes	2.4 (1.2‐4.8)	0.40	4.6 (3.7‐6.0)	<0.01
No	1.8 (1.0‐4.0)		5.3 (4.2‐6.5)	
Criterion III				
Yes	2.8 (1.5‐4.9)	0.14	4.7 (3.6‐6.0)	0.01
No	1.8 (1.1‐4.1)		5.1 (4.1‐6.3)	
Criterion IV				
Yes	2.1 (1.1‐4.4)	0.65	4.5 (3.7‐5.8)	<0.01
No	2.0 (1.1‐4.4)		5.4 (4.2‐6.5)	
Criterion V				
Yes	2.2 (1.2‐4.7)	0.13	4.7 (3.9‐6.0)	<0.01
No	1.9 (0.9‐4.3)		5.3 (4.2‐6.5)	

CRP, C‐reactive protein; MetS, metabolic syndrome [Criteria: I. Waist circumference >102 cm (>40 in); II. HDL cholesterol levels <40 mg/dL (<1.0 mmol/L), or being actively treated for low HDL levels; III. Serum triglycerides levels ≥150 mg/dL (≥1.7 mmol/L), or being actively treated for elevated triglycerides; IV. Fasting glucose levels ≥100 mg/dL (≥6.1 mmol/L), or being actively treated for hyperglycaemia, and; V. Diagnosis of elevated blood pressure or being actively treated for hypertension].

Values express median and interquartile range. Statistical test *t*‐Student.

### 3.3 Influence of MetS, CRP and testosterone levels in the diagnosis of PCa

**Figure 1:**
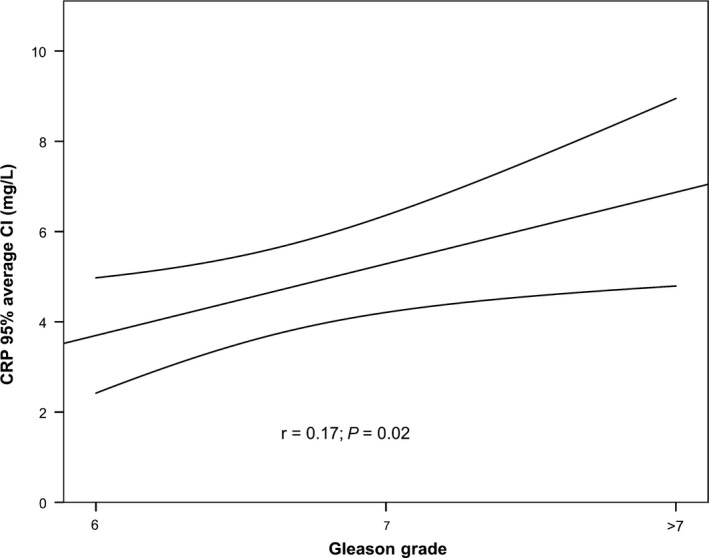
Correlation curve between circulating CRP levels and Gleason Score in patients with PCa

We next analysed the influence of: (a) the MetS status; (b) each individual criterion of MetS; (c) the number of MetS criteria met and (d) circulating CRP or testosterone levels, on the diagnosis of PCa or Sig‐PCa (GS ≥7) (Table 3). Specifically, we found that a greater number of MetS criteria tended to be associated with a higher risk of PCa (*P* = 0.07; being a higher blood pressure the only criteria significantly associated with the risk of PCa; Table 3). Interestingly, we found that the presence of MetS, a greater number of MetS criteria, and higher circulating CRP (but not testosterone) levels were significantly associated with a higher risk of Sig‐PCa. Moreover, when we analysed each MetS criterion independently, we found that only criteria I (waist circumference) and V (elevated blood pressure) were associated with higher risk of PCa (although only a trend was found for Criteria I; *P* = 0.07; Table 3), as well as with higher risk of Sig‐PCa (Table 3). However, no association was observed between criterion I or V and GS (data not shown). Furthermore, it should be mentioned that although a strong correlation between BMI and waist circumference was observed in our cohort, we did not found any association between BMI and the risk of PCa or Sig‐PCa. On the basis of these results, we next analysed whether a greater number of MetS criteria or the circulating levels of CRP were associated to GS in PCa patients. Interestingly, our results revealed that only a higher circulating CRP levels, but not number of MetS, was positively correlated with a higher GS (GS = 6, GS = 7, GS >7; *P* < 0.05; Figure 1).

**Table 3:** Univariate analysis showing the influence of MetS, circulating C‐reactive protein or testosterone levels on the diagnosis of PCa, and clinically significant PCa (Gleason Score ≥7)

Variable	PCa, n = 195			PCa, Gleason ≥7, n = 97		
	OR	*P*	95% CI (OR)	OR	*P*	95% CI (OR)
MetS (yes)	1.17	0.39	0.82‐1.66	1.62	0.03	1.04‐2.54
No. of MetS criteria	1.13	0.07	0.99‐1.29	1.23	0.02	1.04‐1.45
MetS criteria						
Criterion I vs no MetS	1.39	0.07	0.97‐1.98	1.71	0.02	1.08‐2.72
Criterion II vs no MetS	0.96	0.83	0.67‐1.37	1.06	0.78	0.68‐1.65
Criterion III vs no MetS	1.05	0.84	0.69‐1.58	1.23	0.41	0.75‐2.02
Criterion IV vs no MetS	1.13	0.49	0.79‐1.60	1.20	0.41	0.77‐1.87
Criterion V vs no MetS	1.60	0.01	1.13‐2.29	1.76	0.02	1.13‐2.79
CRP (mg/L)	1.02	0.11	0.99‐1.05	1.04	0.02	1.01‐1.07
Testosterone (ng/mL)	0.93	0.15	0.85‐1.02	0.96	0.48	0.85‐1.08

CRP, C‐reactive protein; OR, odds ratio; PCa, prostate cancer; MetS, metabolic syndrome [Criteria: I. Waist circumference >102 cm (>40 in); II. HDL cholesterol levels <40 mg/dL (<1.0 mmol/L), or being actively treated for low HDL levels; III. Serum triglycerides levels ≥150 mg/dL (≥1.7 mmol/L), or being actively treated for elevated triglycerides; IV. Fasting glucose levels ≥100 mg/dL (≥6.1 mmol/L), or being actively treated for hyperglycaemia, and; V. Diagnosis of elevated blood pressure or being actively treated for hypertension].

Further exploratory analyses were carried out to evaluate the association of drug intake or levels of HbA1c, with the diagnoses of both PCa and Sig‐PCa. Specifically, no significant association between HbA1c levels or statin intake and the diagnoses of PCa or Sig‐PCa was observed in our cohort of patients. However, the analysis of metformin intake revealed a significant association with an increased risk of Sig‐PCa even when adjusting by glucose levels and HbA1c (odds ratio [OR]: 2.74 [1.41‐5.31]; *P* < 0.01).

### 3.4 MetS, CRP and testosterone levels as predictive factors of PCa

On the basis the previous results indicating the association between a higher risk of Sig‐PCa with the diagnoses of MetS, a greater number of MetS criteria and higher circulating levels of CRP, we next implemented a multivariable analysis to determine the strength of the independent association of these factors with the risk of being diagnosed with a Sig‐PCa. To that end, a ROC curve analysis was firstly performed to determine the best CRP cut‐off levels for the diagnosis of Sig‐PCa, which revealed that the best value was 2.5 mg/L for CRP (AUC 0.60; *P* = 0.003).

**Figure 2:**
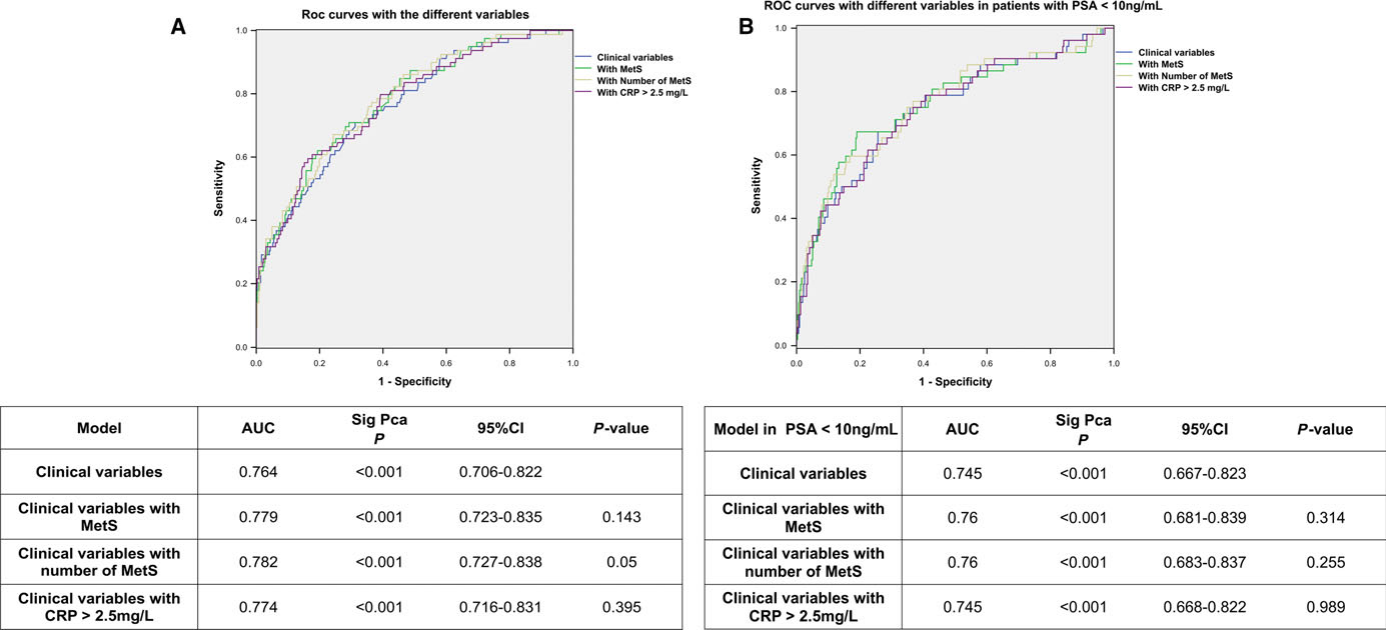
ROC curves showing the predictive ability of different variables (Presence of MetS, number of MetS criteria or circulating CRP levels) to predict a higher risk of significant PCa (Sig‐PCa) when are added to risk factors; age, family History, PSA, 5α reductase inhibitors intake, DRE, prostate volume and number of biopsies; (A) within the total cohort (n = 441 patients; PCa Gleason ≥7 = 79). (B) In patients with PSA <10 ng/mL (n = 368 patients; PCa Gleason ≥7 = 52) (for this analysis the PSA was not categorized and was evaluated as a continuous variable). *P*‐value represents the comparison of each ROC curve with the basal ROC curve with the clinical variables alone

It should be mentioned that, as might be expected, a significant association was observed between the risk of detecting a higher rate of Sig‐PCa in our cohort of patients and an older age (ie <60 vs 60‐70, or vs >70 years old), an elevated PSA levels (ie <3 vs 3‐10, vs 10‐20, or vs >20 ng/mL) or, an abnormal DRE (Table 4). Conversely, this risk significantly decreased in those patients who had a larger prostatic volume and a previous negative biopsy. Therefore, based on these associations, and to accurately determine whether the presence of MetS, a greater number of MetS criteria, or circulating CRP levels might be used as predictive factors of Sig‐PCa independently, we adjusted these three variables by age, family history, PSA, 5α reductase inhibitors intake, DRE, prostate volume and number of biopsies (Table 5). Remarkably, we found that the three variables analysed were significant associated with a higher risk of Sig‐PCa as follow (Table 5): (a) Presence of MetS (OR: 1.83, 95% CI: 1.05‐3.20, *P* = 0.03); (b) number of MetS criteria (OR: 1.30, 95% CI: 1.05‐1.60, *P* = 0.02); and, (c) circulating CRP levels (>2.5 mg/L; OR: 2.00, 95% CI: 1.14‐3.51, *P* = 0.02). In fact, ROC curve analyses confirmed that the presence of MetS, a greater number of MetS criteria, or circulating CRP levels might be used as additional diagnostic factors for Sig‐PCa when are added to the common risk factors mentioned above (ie age, family history, PSA, 5α reductase inhibitors intake, DRE, prostate volume and number of biopsies) with an AUC of 0.78 (0.72‐0.84), 0.78 (0.73‐0.84) and 0.77 (0.72‐0.83) respectively (Figure 2A). It should be mentioned that the combination of these three clinical variables together did not significantly increase the predictive ability of the diagnosis of Sig‐PCa (Figure 2A). However, a clear trend was found to diagnose Sig‐PCa when adding the number of MetS, which might justify future evaluations in higher cohorts. Furthermore, in a sub‐analysis in patients with a PSA<10 ng/mL, the AUC only showed a non‐significant increase with the addition of the presence of MetS or the number of MetS criteria, but not with CRP levels (AUC of 0.76 vs 0.745) (Figure 2B).

**Table 4:** Univariate analysis of common predictive factors of significant PCa on biopsy

Variables	Sig‐PCa (Gleason ≥7)		
	OR	*P*	95% CI (OR)
Age 60‐70 vs <60 (years old)	1.66	0.10	0.90‐3.07
Age >70 vs <60 (years old)	5.35	<0.01	2.87‐9.98
PSA 3‐10 vs <3 (ng/mL)	2.67	0.07	0.93‐7.66
PSA 10‐20 vs <3 (ng/mL)	5.42	<0.01	1.70‐17.34
PSA >20 vs <3 (ng/mL)	30.44	<0.01	8.73‐106.11
DRE (suspicious)	3.70	<0.01	2.29‐5.99
Prostate volume (cc)	0.98	0.02	0.97‐0.99
Number of biopsy >1 (yes)	0.34	<0.01	0.18‐0.66
Family history (yes)	0.74	0.35	0.39‐1.39

DRE, digital rectal examination; PCa, prostate cancer; OR, odds ratio.

PSA ‐ Adjusted by 5‐α reductase inhibitors. [Prostate volume (N = 441 patients; PCa Gleason ≥7 = 79)].

**Table 5:** Multivariate analysis of the predictive ability of different variables (presence of MetS, number of MetS criteria or circulating CRP levels) to predict a higher risk of Sig‐PCa adjusting by age, PSA, 5‐α reductase inhibitors intake, DRE, prostate volume and number of biopsies

	Multivariate analysis of MetS			Multivariate analysis of number of MetS criteria			Multivariate analysis of CRP levels		
	OR	*P*‐value	95% CI (OR)	OR	*P*‐value	95% CI (OR)	OR	*P*‐value	95% CI (OR)
Age 60‐70 vs <60 (years old)	1.74	0.14	0.83‐3.68	1.97	0.20	0.77‐3.49	1.81	0.12	0.86‐3.83
Age >70 vs <60 (years old)	4.78	<0.01	2.14‐10.66	4.55	<0.01	2.04‐10.18	5.04	<0.01	2.25‐11.30
PSA 3‐10 vs <3 (ng/mL)	2.64	0.09	0.85‐8.19	2.66	0.09	0.85‐8.28	2.42	0.12	0.79‐7.44
PSA 10‐20 vs <3 (ng/mL)	4.99	0.02	1.34‐18.64	5.07	0.02	1.36‐18.98	3.98	0.03	1.07‐14.77
PSA>20 vs <3 (ng/mL)	19.69	<0.01	4.36‐88.97	20.76	<0.01	4.57‐94.28	13.94	<0.01	3.09‐62.90
5‐α reductase inhibitors intake	1.19	0.79	0.32‐4.31	1.21	0.77	0.33‐4.40	1.31	0.68	0.36‐4.81
DRE (suspicious)	1.59	0.15	0.85‐3.01	1.61	0.14	0.85‐3.04	1.79	0.08	0.94‐3.42
Prostate volume (cc)	0.98	<0.01	0.96‐0.99	0.98	<0.01	0.96‐0.99	0.98	<0.01	0.96‐0.99
Number of biopsy >1 (yes)	0.32	<0.01	0.13‐0.76	0.32	0.01	0.13‐0.77	0.36	0.02	0.15‐0.84
Family history (yes)	1.19	0.64	0.56‐2.57	1.25	0.57	0.58‐2.68	1.14	0.74	0.53‐2.42
MetS (yes)	1.83	0.03	1.05‐3.20						
No. of MetS criteria				1.30	0.02	1.05‐1.60			
CRP >2.5 mg/L							2.00	0.02	1.14‐3.51

## 4 DISCUSSION

MetS and PCa are highly prevalent conditions worldwide. Current evidence suggests that MetS could play a role in the development and progression of several neoplasms, including PCa.^6^, ^21^ However, the specific components of MetS that may contribute to PCa risk and progression/aggressiveness in human remains controversial. In this sense, we have previously demonstrated the existence of a tight cross‐talk between the metabolic status and the development and homoeostasis of the prostate gland, wherein key metabolic components (eg insulin, leptin, etc.) could play a relevant pathophysiological role at the prostate level.^7^, ^8^ Moreover, it has been shown that androgen‐deprivation therapy in patients with PCa results in changes that overlap with MetS, including decreased insulin sensitivity, increased triglycerides and increased fat mass.^22^ Despite the efforts and progresses made in recent years, it is imperative to determine the real impact of MetS, and/or of its individual components on PCa development, as well as the to determine the risk factors that comprise MetS in men with PCa to treat them accordingly.

In this study, we aimed at determining the potential associations and clinical consequences that MetS, each of the individual criterion of MetS, circulating testosterone levels and inflammatory status (using circulating CRP levels) may have on the risk and aggressiveness of PCa. As previously reported,^16^, ^23^, ^24^ we observed an association between MetS and/or most of its individual criterion with lower circulating levels of testosterone and higher circulating levels of CRP. Furthermore, the analysis of the different clinical characteristics comparing patients with and without MetS revealed that patients with MetS had slightly higher prostate volume compared with patients without MetS, which is consistent with a recent report indicating an association of MetS parameters with benign prostatic enlargement in men surgically treated for this pathology.^25^ These data might suggest that some component of the MetS could be connected with the prostatic growth and, therefore, given that the prevalence of MetS is increasing worldwide, the clinical control of MetS should be considered in patients at risk of PCa.

In line with this, the majority of the previous studies analysing the association between MetS and PCa have used the definition established by the NCEP ATP III,^21^ which have often obtained inconsistent conclusions, probably due to the fact that the individual diagnostic criterion of MetS have not been consistently and uniformly examined in these studies.^21^, ^26^, ^27^, ^28^, ^29^, ^30^, ^31^, ^32^, ^33^, ^34^, ^35^, ^36^, ^37^, ^38^, ^39^ In contrast, in this study, we have analysed the presence of MetS and of each MetS criterion independently using a significant cohort of patients (n = 524) with and without MetS, and with and without PCa (n = 240 with MetS [94 with and 146 without PCa] and n = 284 without MetS [101 with and 183 without PCa]). Remarkably, we found that the rate of Sig‐PCa diagnoses was significantly higher in patients with MetS compared with patients without MetS (*P* = 0.03). Furthermore, our study indicated that the presence of MetS as well as a greater number of MetS criteria was significantly associated with a higher risk of Sig‐PCa. In fact, multivariate analysis ROC curve analyses revealed that the presence of MetS and a greater number of MetS criteria could be used as diagnostic factors for Sig‐PCa. Consistent with our study, Bhindi et al,^28^ who previously investigated the criteria of MetS as quantitative variables, also observed that the greater the number of MetS criteria met, the greater the risk that patients had of harbouring a Sig‐PCa. Interestingly, when we analysed each MetS criterion individually, we found that a higher waist circumference and elevated blood pressure (criteria I and V, respectively) were the only two factors significantly associated with an increased risk of PCa and of Sig‐PCa in our cohort of patients, which is further supported by previous meta‐analysis published on this specific topic.^40^ In this sense, it should be mentioned that, although BMI has been commonly used to define obesity, BMI is probably less precise than the waist circumference which has been shown to have a stronger association with the inflammatory status and cardiovascular risk.^41^ In fact, we found a strong correlation between both BMI and waist circumference in our cohort of patients; however, more individuals were considered as obese patients when waist circumference was used to categorize them vs BMI (ie 55% [waist circumference] vs 33% [BMI]). Furthermore, previous data have showed that waist circumference as a quantitative variable is associated with a higher risk of PCa or Sig‐PCa after adjusting by BMI,^42^ which is further validated in our cohort showing an OR: 1.07 (95% CI: 1.03‐1.12, *P* = 0.002) for Sig‐PCa.

Interestingly, since the use of metformin and statins and the risk of PCa is a controversial topic worldwide,^43^, ^44^, ^45^ we also analysed this association in this study. Specifically, we did not observe an association between metformin or statins intake and the diagnosis of PCa in our cohort of patients; however, a clear association was found between metformin, but not statins, intake and the diagnose of Sig‐PCa. Nevertheless, this observation should be taken with caution since, it was based on an exploratory analysis of drug intake and the presence of PCa at the time of prostate biopsy using a limited number of patients under metformin treatment and, without evaluating the period of time under the drug intake (which was not available in our cohort), being this latter parameter essential in this analysis since evidences have showed that only those patients with long‐term consumption of metformin are the patients with less risk of any PCa.^44^

Since the available studies focusing on the association between circulating testosterone levels and the risk of developing PCa are in many instances controversial,^46^, ^47^ we next explored the association and independent predictive ability for Sig‐PCa diagnoses of circulating testosterone levels among patient at risk of PCa and found no association between testosterone levels and an increased risk of PCa or Sig‐PCa on the prostate biopsy in our cohort of patients. In contrast, we observed a clear association between elevated circulating CRP levels and a higher risk of Sig‐PCa. Moreover, multivariate analysis showed that circulating CRP levels could be used as diagnostic factor for Sig‐PCa. These observations are in part consistent with some, but not all^48^, ^49^, ^50^, ^51^, ^52^, ^53^, ^54^ early reports showing that circulating CRP levels are associated with the prognosis of PCa (advanced and metastatic disease). Of note, our results also revealed that higher circulating CRP levels were associated with PCa aggressiveness since its circulating levels were positively associated with higher GS in our cohort of PCa patients. To the best of our knowledge, this is the first report showing that baseline circulating levels of CRP are associated with a higher risk of detecting PCa at the time of biopsy and demonstrating that circulating CRP levels could be used as a putative biomarker of PCa aggressiveness.

Finally, it should be mentioned that some observations reported in this study might have certain limitations and therefore, should be interpreted with some caution. First, although the use of TRUS biopsy for PCa diagnosis suffers from random error and false negative results in comparison with trans‐perineal template biopsy, which might have affected the results of this study, it should be emphasized that TRUS biopsy is worldwide spread and the standard method in the current clinical practice.^55^ Likewise, it would be preferable to have compiled data of multiple CRP and testosterone levels from each patient over a larger time interval rather than a single value. Finally, the onset of MetS from diagnosis in each patient would ideally have been recorded as well to determine if the chronicity of the disease influences the degree of observed inflammation, and CRP levels. Nonetheless, based on the high incidence of MetS worldwide, especially in western countries, and considering the evident connection between some of the components of the MetS and the risk of PCa at the time of prostate biopsy, as well as of the association between inflammatory status with the aggressiveness of PCa found in our study, the results of the present work invites to suggest that interventional studies based on the control of MetS and inflammatory status in patients at risk of PCa might be a key point in the overall management of this disease. Therefore, future cellular/molecular/translational studies are crucial to understand the specific connections between individual MetS determinants and the pathophysiology of PCa.

## AUTHOR CONTRIBUTION

EG‐G, JC‐V, RML and MJR‐T conceived and designed the project; EG‐G, JC‐V, JPC‐H, AMB‐P, JV‐R, JR‐G and MJR‐T acquired data; EG‐G, JC‐V, AMB‐P, JMJ‐V and RML performed the analysis and interpretation of data; EG‐G, JC‐V and RML wrote the manuscript; JPC‐H, AMB‐P, JMJ‐V, JV‐R, JR‐G and MJR‐T revised the manuscript for important intellectual content; EG‐G, JMJ‐V performed the statistical analysis and imaging; RML and MJR‐T obtained funding; RML supervised the work.

## CONFLICTS OF INTEREST

Nothing to declare.
